# Association of neural tube defects with congenital abnormalities of the urogenital system in a Chinese cohort

**DOI:** 10.1186/s12887-021-02492-8

**Published:** 2021-02-05

**Authors:** Zhi-Hua Hong, Dong-Hui Jin, Xiao-Jian Yuan, Yang Zhao, Hou-Wei Lin, Jie Chen

**Affiliations:** 1 Department of Pediatric Surgery, Jiaxing Maternity and Child Health Care Hospital, No. 2468, Zhonghuan East Road, Nanhu District, 314051 Jiaxing, China; 2 Department of Pediatric Surgery, Xinhua Hospital, School of Medicine, Jiaxing Branch, Shanghai Jiaotong University, 314051 Jiaxing, China

**Keywords:** Neural tube defect, Congenital, Genitourinary urogenital malformation, Incidence, Retrospective analysis

## Abstract

**Background:**

This study aimed to retrospectively analyze the correlation between congenital abnormality of the urogenital system and various factors in children with neural tube defects (NTDs).

**Methods:**

A total of 190 children with congenital NTDs, who were admitted to a hospital from May 2013 to May 2018, were included into the present study. All admitted children with congenital NTDs were carried out routine abdominal B-ultrasound examinations to determine the malformations of the abdominal organs, including the urinary system. Children with a B-ultrasound result of suspected and unsure malformation underwent intravenous pyelography (IVP) and voiding cysto-urethrography (VCU), and this was further confirmed by the CT results.

**Results:**

The incidence of urogenital malformation was 12.1% (23/190) in children with congenital NTDs. For the 23 children with urogenital malformations, most of these children had no definite urinary system symptoms, while some of these children had multiple incidences of urinary system infections.

**Conclusions:**

Congenital NTDs are often combined with urogenital malformations, if not specifically searched these may be overlooked. The early detection of these malformations is beneficial to reduce the risk of operation and improve the prognosis.

## Background

Neural tube defects (NTDs), which are also known as dysrhaphia, are congenital malformations caused by the failure of closure of the neural tube during its development. This can be divided into two types: open type and closed type [1]. Congenital NTDs are often combined with other systemic malformations. Among these, urogenital malformation is one of the malformations with the highest incidence [2]. Hunt and Whitaker in 1987 and Sally-Anne Hulton in 1990 reported that the incidence of renal malformation in children with congenital NTDs was 8.9% and 10.4% [3, 4], respectively. This has aroused the clinical attention of researchers to congenital NTDs combined with urogenital malformation. The early detection of concomitant malformations and the development of an appropriate treatment plan would not only help to reduce the potential risk of surgery, but also help to improve the long-term prognosis of children [5].

During development, the neural tube originates from mesoderm cells, and begins in the third week of embryo. The main organs of the urinary system and reproductive system originate from the mesoderm, and develop to the urogenital ridge from the 3rd to the 7th week of embryo development [6]. Based on embryology, Neidhardt et al. [7] proposed a hypothesis that malformation is induced by the effect of teratogenic factors on the differentiation of the mesoderm in early embryo. This provides an embryological basis for congenital NTDs combined with other malformations, especially those of the urogenital system from the same mesoderm, with a similar differentiation period [8].

The causes of NTDs include genetic factors, decreased production of chorionic gonadotropin in early pregnancy or the insensitivity of embryonic receptor cells to the hormone, deficiency of vitamin B12 and folic acid, and abnormal internal environment in pregnancy such as diabetic ketoacidosis. This suggests that NTDs are correlated to both genetic and environmental factors [9]. The causes of congenital malformation of the urogenital system are complex, and there is no final conclusion on which factors have a direct impact on this [10].

Therefore, the present study was designed to investigate the incidence of urogenital malformation in children with congenital NTDs, analyze its clinical and imaging characteristics, and explore the correlation between this and various factors, in order to provide ideas for the prevention and treatment of urogenital malformation in children with congenital NTDs.

## Information and Methods

### Methods

A total of 190 children with congenital NTDs, who were admitted to the Neurosurgery Department of our hospital (shanghaixinghua hospital) from May 2013 to 2018, were retrospectively analyzed. The general clinical data of all children were recorded in detail, which included their age, gender, place of residence, birth order, age of the parents at birth, and previous medical history.

All children underwent routine abdominal B-ultrasound at admission to determine the condition of the abdominal organs, including the urogenital system, received routine X-ray, computed tomography (CT) and magnetic resonance imaging (MRI) to identify the NTDs, and received an electrocardiogram to determine whether the heart was abnormal. If the patient has a positive result or a suspected positive result, an echocardiography was performed for diagnosis.

Since May 2013, the Neurosurgery Department of our hospital began to carry out routine abdominal B-ultrasound examinations for all admitted children with congenital NTDs, in order to determine the malformations of the abdominal organs, including the urinary system. Children with a B-ultrasound result of suspected and unsure malformation underwent intravenous pyelography (IVP) and voiding cysto-urethrography (VCU), and this was further confirmed by the CT results [11].

Inclusion criteria: children with definitely diagnosed NTDs, and children with complete clinical, laboratory and imaging data.

Exclusion criteria: children with NTDs, and simple neurobladder and hydronephrosis indicated by ultrasonography, and children without imaging data.

###  Statistical analysis

The data were statistically analyzed using statistical software SPSS 11.5. Chi-square test, rank sum test and multivariate correlation analysis were performed for the analysis. *P* < 0.05 was considered statistically significant.

## Results

###  General data

Among the 190 children, 100 children were boys and 90 children were girls, and the age of these children ranged from five days old to 17 years old, with an average age of 1.48 years old.

### Types and incidence of congenital abnormalities of the urogenital system

Among the 190 children, 56 children were complicated with malformations of other systems (Tables 1 and 2) due to multiple malformations, and the total number of patients with malformations was 63.

**Table 1:** Characteristics of different types of NTDs complicated with urogenital system abnormality

	Open NTDs	Closed NTDs
Number of cases (%)	82/190 (43.2% )	108/190 (57.8%)
Female (%)	47/82 (58.3%)	43/108 (39.8%)
Male (%)	35/82 (42.7%)	65/108 (60.2%)
With genitourinary system abnormality (cases,%)	10/82 (12.2%)	13/108 (12%)

**Table 2:** Complications of genitourinary system abnormality and other system malformations

Other system malformations	Genitourinary system abnormality	
	Positive	Negative
	30	160
Cardiovascular system	4	10
Gastrointestinal tract	2	4
Musculoskeletal system^a^	4	2
Ent and oral	0	2
Central nervous system	1	4

^a^Except for the hoof foot, varus foot and high arch foot secondary to tethered spinal cord

As presented in Tables 3 and 23 children were complicated with urogenital malformations, with an incidence was 12.1% (23/190), and the total number of urogenital malformations was 30. Solitary kidney and renal dysplasia were the most common, and both accounted for 16.7% (5/30) of the total urogenital malformations. These were followed by renal ureteral duplication (13.3%, 4/30), horseshoe fusion kidney (3%, 4/30), hypospadias (13.3% 4/30), ectopic kidney (10%, 3/30), cryptorchidism (10%, 3/30), bladder ectropion (3.3%, 1/30), and bladder duplication (3.3%, 1/30).

**Table 3:** Number and proportion of various genitourinary malformations

Malformed categories	Proportion(%)
Solitary kidney	5 (16.7%)
Renal dysplasia	5 (16.7%)
Renal ureteral duplication	4 (13.3%)
Horseshoe fusion kidney	4 (13.3%)
Ectopic kidney	3 (10%)
Bladder duplication	1 (3.3%)
Bladder ectropion	1 (3.3%)
Hypospadias	4 (13.3%)
Cryptorchidism	3 (10%)

### Urinary system symptoms

For the 23 children with urogenital malformations, most of these children had no definite urinary system symptoms, while some of these children had multiple incidences of urinary system infections. Furthermore, one child had bladder ectropion, four children had hypospadias, and three children with cryptorchidism were detected with a malformation due to the abnormal appearance in the early stage. In the treatment of malformations, children with bladder ectropion, bladder duplication, hypospadias and cryptorchidism underwent surgical therapy during their childhood, while the urogenital malformations of the remaining children were not treated, and continued to receive a follow-up examination in the Urology Department.

### The correlation between congenital abnormality of the urogenital system and various factors

The statistical analysis revealed that the differences in gender (*P* = 0.264), birth place (urban or rural, *P* = 0.445), birth order (*P* = 0.478), age of the mother (*P* = 0.148) and NTD type (*P* = 0.324) between children with and without urogenital malformation were not statistically significant. However, children with urogenital malformations were prone to skeletal muscle system malformation (*P* = 0.012).

## Discussion

The incidence of urogenital malformations in the normal population was compared [9]: 0.07% for solitary kidney, 0.003% for renal dysplasia, 0.25% for horseshoe fusion kidney, and 0.8% for duplication and 0.05% for ectopic kidney. In the present study, the incidence of urogenital malformation in children with congenital NTDs was significantly higher than that in normal subjects. This suggests that urogenital malformation in children with congenital NTDs remains as a clinical problem that needs to be urgently solved.

Previous researches show that NTDs were caused by genetic factors, decreased production of chorionic gonadotropin in early pregnancy or the insensitivity of embryonic receptor cells to the hormone, etc. [12], indicating that NTDs are correlated to both genetic and environmental factors [13]. The incidence of urogenital malformation in the present study was lower than that reported in foreign literatures. The reason may be due to regional and ethnic differences. Among all malformations, solitary kidney had the highest incidence. This is similar to the reports of foreign literatures. The differences in gender, birth place (urban or rural), birth order, age of the mother and NTD type between children with and without urogenital malformation were not statistically significant. Furthermore, the present study revealed that children with urogenital malformation are prone to skeletal muscle system malformation (*P* = 0.012). Therefore, in the perspective that children with urogenital malformations are prone to skeletal muscle system malformation, urogenital malformation may be the most closely related to genetic factors. This also explains why urogenital malformations are not correlated to environmental factors in the present study. Similar to previous studies, unilateral renal agenesis will not increase the chance of disease of the contralateral kidney. However, if the patient develops a disease, the prognosis will be poorer than those with two normal kidneys [12]. This explains the reason for the correlation between these malformations. In addition, ectopic kidney can cause hydronephrosis and lithogenesis due to the abnormal position of the kidney, and induce renal hypertension [12, 13]. Furthermore, although renal dysplasia is often asymptomatic, hypertension can be caused by vascular malformation [13]. in the process of embryonic development, the skeletal muscle system was also homologous with the urogenital system that originated from the mesoderm, and the development time is also in the early embryo [14–18].

In the present study, among the 30 children with urogenital malformation, merely six cases were diagnosed due to the abnormal appearance in early stage, while the remaining cases had no clinical symptoms. The presence of urogenital malformation was merely found during the routine B-ultrasonic examination before the operation. Hence, there is a need for neurosurgeons and urologists to comprehensively evaluate these children, and inform their families of their condition and prognosis. As shown in previous studies, for children with bladder ectropion, bladder duplication, hypospadias and cryptorchidism, it’s suggested to give them surgical therapy during their childhood [18]. Although these urogenital malformations did not progress and no complications occurred during the follow-up, long-term prognoses are needed through further follow-ups.

## Conclusions

Congenital NTDs are often combined with other system malformations, and urogenital malformation is one of the malformations with the highest incidence. The early detection of these malformations is beneficial to reduce the risk of operation and improve the prognosis. Enough attention need to be given to this in clinic, and appropriate examination methods should be used to early detect and treat these malformations.

## Data Availability

Not applicable.

## Abbreviations

NTDs: Neural tube defects
CT: Computed tomography
MRI: Magnetic resonance imaging
IVP: Intravenous pyelography
VCU: Voiding cysto-urethrography
